# Abstracts and Gleanings

**Published:** 1883-11-20

**Authors:** 


### ABSTRACTS AND GLEANINGS.

**Influence of Calomel on Fermentation and the Life of Micro-Organisms.**—The following is an abstract of a paper by N. P. Wassilieff, in New Remedies: Calomel has always held a foremost place amongst those remedies which are confidently resorted to in certain gastric and intestinal disorders, especially of childhood, but the precise nature of its beneficial effect has heretofore been unexplained. Recent works on pharmacology pass over the question, and only Kohler refers to the favorable action of the drug in typhus, cholera, dysentery and other diseases, as being due to the germicidal and anti-fermentative qualities. No evidence in support of this view is adduced. Voit, however, had noticed, in 1857, that egg-albumen and blood, when mixed with calomel, remained for days without undergoing putrefaction. Hoppe-Seyler also mentions an aseptic influence of calomel, and ascribes to it the well-known color of bowel discharges after an administration of calomel.

The author undertook this investigation at the request of Hoppe-Seyler, first, in regard to the behavior of calomel towards the so-called unorganized fluids (enzymes), and, secondly, as to its action on the lower organisms associated with the processes of fermentation and putrefaction. The first series of experiments were made in order to determine the influence of calomel on the normal process of digestion in the stomach. The results proved that its presence in no way interfered with the properties of the gastric juice, fibrin being digested in the same time, whether calomel was present or not. In the next series, the influence of calomel on the process of pancreatic digestion was investigated. It is now known that three separate ferments exist in the pancreatic secretion, by which albuminates, fats and carbo-hydrates are severally transformed and fitted for assimilation in the system. The object in view was to observe the possible influence of calomel on each of these respective ferments. For the purpose of experiment, a watery extract was prepared from the finely-minced gland and strained through linen. It was found that the action of the ferment, by which albuminates become digested, was in no respect hindered by the presence of the calomel, and further, that there was a conspicuous absence from the liquid mixture of all products of putrefaction. In the mixture containing calomel large quantities of leucin and tyrosin were found, whilst indol and phenol were absent. In the mixture without the addition of calomel, the two latter bodies were both present, but only traces of leucin and tyrosin. The latter solutions had likewise a putrid smell and a dirty-brown color, whilst the former was of a dark-gray color, and odorless. In some additional experiments wherein the process was allowed to proceed in a Bunsen gasometer, and the evolved gases examined; it was found that from the mixture containing calomel, hydrogen and hydrogen-sulphide were never given off, and carbonic anhydride in very considerably less amount than from the control mixtures without calomel. These results accord with those of Hufner (J. pr. Chem., 10 and 11), who found, in his experiments on artificial digestion with pancreatic extract, that when, by means of a properly arranged apparatus, entrance of micro-organisms was prevented, neither hydrogen nor hydrogen-sulphide made its appearance, but only carbonic anhydride. These two first-named gases have, therefore, nothing to do with digestion proper, but are the result of putrefactive changes brought about by the presence of microzymes in the alimentary canal.

The action of calomel on the ferment of the pancreatic juice, to which the digestion of fat is due, was next examined. The existence of such a principle has, until now, been considered highly doubtful, Paschutin's observations on this head being all that is known of the subject (W. Paschutin, *Ueber Trennung der Verdauungs-Fermente; Centralbi fur die Medicin. Wissensch, 1882*). As in putrid solutions, fats became saponified rather quickly; the problem became an important one to determine whether the transformation of fat in the alimentary canal was owing to the action of an unorganized ferment or merely to the putrescent changes going on there. The experiments made proved beyond a doubt that the action of pancreatic juice upon fat took place in the complete absence of putrefaction, and the digestion of the fat by the pancreatic extract in presence of calomel proceeded precisely as in the instance of the èxperiments in regard to the peptic ferment (trypsin) of that gland. The action of the third and remaining ferment of the pancreas, the diastatic, upon starch, and the transformation of the latter into glucose, proceeded equally undisturbed in the presence of calomel. Hence it follows that calomel, by its presence in these experiments on artificial digestion, allows the actual process of digestion to go on without injury, whilst it effectually prevents putrefactive change. And this, in the same way, is proved for salicylic acid by Kuhn, and, in the case of arsenic, by Scheffer and Böhm.

The author also found the action of calomel in the process of butyric acid fermentation, which sometimes occurs in certain pathological states of the digestive system, similar to that in common putrefaction, entirely preventing it. A further series of experiments, which need only be referred to here, were carried out to determine the disinfectant action of calomel in fluids containing bacteria and micrococci, the bacterioscopic method of Bucholtz-Wernich being used. The results showed that calomel acted as a true antiseptic and disinfectant in preventing the development of such organisms in culture fluids, and arresting their activity when already developed therein. The difference in the influence of calomel on the process of digestion on the one hand and on putrefactive and fermentative changes on the other, is dependent upon a distinct difference of action on unorganized ferments. Whilst it does not interfere with the activity of the latter, it destroys the vitality of the former, and with it the power of inducing subsequent septic changes. Finally, as regards the green color of the bowel discharges witnessed after the exhibition of calomel, this was formerly attributed to the presence of bile expelled by virtue of the assumed action of calomel as a cholagogue, this appearance was considered by Hoppe-Seyler to be due to the presence of undecomposed bile, and the author's expcriments now confirm this view.

Under ordinary conditions, the bile pigments, bilirubin and biliverdin, become decomposed in the intestine under the influence of putrescent changes, forming hydrobilirubin. During the administration of calomel this decomposition does not take place, and the bile pigments are expelled unchanged. The author concludes that the therapeutic virtues of calomel are to be ascribed to its antiseptic and disinfectant properties.—*Zeitsch. Phys. Chem.*, *6*, *112*, *in Journ. Chem. Soc.*

**Benzoate of Cinchonidine.**—This salt is prepared, according to Byasson, in the following manner:

Dissolve 60 parts of benzoic acid in 200 parts of alcohol of 90 per cent., and pour the solution into a porcelain vessel containing 3,000 parts of boiling distilled water. On the other hand, dissolve 200 parts of sulphate of cinchonidine in 2,000 parts of water, with the aid of diluted sulphuric acid and precipitate with ammonia. Wash with a small quantity of cold water. Add the still moist precipitate to the hot solution of benzoic acid, and filter while hot. If the solution is not faintly alkaline, make it so by the cautious addition of ammonia. On cooling, the benzoate of cinchonidine separates in form of small, thin, prismatic needles, much resembling the neutral sulphate of cinchonidine. By evaporation of the mother-water, a new crop of crystals is obtained, which are added to the first, and then washed with cold water. The yield is about 200 parts.

Bouchardat recommends this salt in mild cases of diabetes with excessive uric acid production, which he calls "glyco-polyuric diabetes." He follows the authority of Ure, who states that benzoic acid unites with uric acid, and forms hippuric acid or alkaline hippurates, which are both more soluble than uric acid or urates.—*Pharm. Post*, *No. 34*.

**Treatment of Nævus.**—There are several recognized and legitimate ways of treating "mothers' marks," as nævi or vascular stains are denominated. One method is to remove the discolored integument by a series of elliptical excisions ; and another is to puncture the disorganized skin with a cataract needle ; and still another is to paint the nævoid spot with tincture of thuja.

Lately I have had under my treatment a nævus—arterio venous —of the eyelid. It involved the integument and the conjunctiva, so that excision was impracticable. Besides, the alcohol of the thujal tincture provoked undue irritation of the eye. To hasten a cure I injected a few drops of the tincture of thuja into the vascular mass every week, using a small hypodermic needle for the execution of the purpose. Some inflammation followed the injections, yet this was in no way baleful. In ten weeks no deformity existed. Injections were made on six different occasions. The result was highly satisfactory. Nævi of the vulva may be cured by the same method.—*Ec. Med. Journal.*

**Doctor Kocher,** of Berne, read a paper before the Twelfth Congress of the German Surgical Society, held at Berlin, a few months ago. His subject was "Extirpation of Bronchocele and its Results," in which he suggests that the thyroid gland is a regulator of the cerebral circulation. Sometime after the removal of the gland persistent and progressive amæmia arises, proving, as he thinks, that the gland is a blood producing organ. Anæmia after removal of the spleen is but temporary· The paper was followed by one on the same subject by Dr. Bardelebin, of Berlin, in which he denied the blood-forming functions of the spleen and thyrain gland. He removed both these organs in a dog several years since and observed no evil consequences. He claims that in Dr. Kocher's cases the amæmia would have developed without operation.

Experiments on the lower animals have their value, but deductions from them cannot be regarded as absolute.

Dr. Maas, of Freeburg, presented a third paper on "Bronchocele." In five hundred and twenty-two cases, he operated only twenty-three times. In all the other cases the tumor was diminished by treatment with iodoform. We see how important the subject of goitre is in Central Europe.

Dr. Maas also presented a paper on "Alkaloids of Decomposition," which opens up a curious field of study. He found three alkaloids in decaying animal matter. They were present in great quantity and found as early as 24 hours after death. The first in small dose, induced marked tetanus, and a larger dose caused death in 30 to 40 minutes. The action of the second, resembled that of morphia, while the third was similar to strychnia in its effects. These researches suggested that we may have conditions accompanying wounds that are due to the development of these alkaloids.—*Med. World.*

**Aspidospermo Quebracho.**—Dr. John Fearn, in the California Medical Journal, gives the results of his experience with this respiratory stimulant. He concludes, as a result of his observations, that it stimulates and tones the pneumogastric nerve, and especially the cardiac and pulmonary branches of that nerve. He reports two cases in which he employed the agent. In the first there was severe dyspnœa, an anxious look and cyanotic countenance due to cardiac disease. In the second case the dyspnœa was due to pneumonia. In both cases the exhibition of quebracho was promptly followed by relief to the breathing. He enjoins an intelligent choice of the remedy in individual cases, intimating that its promiscuous empirical use must be attended with frequent failures.—*Ther. Gazette.*

**To Hasten the Action of Quinine.**—It is claimed that the administration of a weak tartaric acid lemonade, immediately before swallowing powder or pills of quinine, will facilitate the absorption of that drug. In addition to its effect in thus accelerating solution and absorption, it is said, also, to obviate the unpleasant gastric irritation experienced in the case of some after the administration of large doses of quinine.—*Ther. Gazette.*

**A Remarkable Case of Obstetrics.---Abortion at Two Months and Quadruplets at Full Time.**—Drs. Edwards and McTaggart, in a report (Canada Medical Record), say: On July 21, 1883, we were called to Mrs. S., small of stature, aged thirty-eight, weight one hundred pounds. She thought herself but five months *enciente*, but from hei history and condition it was evident that she was seven months pregnant. She last menstruated on December 4, 1882. About seven weeks from this time she commenced to flow, which lasted for some three weeks, accompanied by pain. With a pain resembling a labor pain something was expelled, which she described as a lump of flesh with bloodvessels in it. To this "lump" was attached a short string. At this she became alarmed, and consulted a medical man, who assured her that she had had a miscarriage. He prescribed some medicine which he said would check the flow and cause the expulsion of anything that might remain. From her account the flow increased for a few days, then finally stopped. From this time until Friday, the 14th September, 1883, she has been, comparatively speaking, quite well, although distressed by the immense size and weight of the abdomen. On the evening of this date (Friday, 14th September) she was delivered of four living children, two boys and two girls; the time elapsing between the birth of the first and that of the last child being one hour and forty-five minutes. The weight of the male children exceeded that of the females by a few ounces. Weight of males, four pounds, nine ane one-quarter ounces, and four pounds three ounces; females, four pounds six ounces, and three pounds, thirteen and three-fourths ounces. Labor terminated faborably, there being no hemorrhage to speak of. There was but one placenta, each cord being inserted at different parts of its surface. The quartette are now six days old, all healthy, able to nurse, and bid fair to live. The mother is doing exceedingly well, having suffered no more exhaustion than if she had had but one child.—*Can. Med. Record.—Louisville Med. News.*

**Hydrastis in Gonorrhœa.**—Dr. A. W. Bixbey reports several cases of gonorrhœa treated chiefly by hydrastis. One of the injections suggested is:

| | | |
|---|---|---|
| ℞ | Hydrastiæ sulphatis | grs. x. |
| | Glycerinæ | 1 ℥ |
| | Aquæ destillat | ℥ iij. |

M. Use by injection every three hours.—*Quarterly Epitome Prac. Med. and Surg.*

**Napthalin in Frostbites.**—Dr. Linderbaum has employed this remedy with success in a number of cases of frostbite. The dressing is usually changed every seven to ten days. In some instances the patients complained for two or three hours after the application of severe stinging pains, caused probably by small crystals of naphthaline. As for us, the author's experience will permit him to judge, the same remedy is equally beneficial in burns—*St. Petersburg Med. Woch.*

**Cancer Remedy.**—A correspondent of the British Medical Journal (No. 1175) reports that great relief has been experienced from the use of "clivers" (Galium aparine) as a remedy for cancer. When applied locally, it reduces the size and diminishes the pain of cancer. In Hertfordshire it is also used internally, the directions being as follows: The bowels having been previously cleared by aperient medicine and the patient enjoined to live on most simple diet, five ounces of the juice of the plant (obtained by pounding and squeezing) are to be taken twice daily; at the same time an ointment of the juice is to be applied to the cancerous ulcer, laying the bruised plant over it, and keeping the dressing constantly applied and frequently renewed. The amendment is very gradual, so that steady perseverance in the use of both internal and external means is necessary. According to one account, in three months the ulcer had perfectly healed.—*Med. Times.*

**Dr. Martinet** thus concludes a paper in a French medical journal on Typhoid Fever during pregnancy:

1. Typhoid Fever is rare in pregnant women.
2. It determines abortion in about one-half of the cases; the more surely, the less advanced is the pregnancy.
3. The lightest forms may produce abortion.
4. This complication arises usually in the course of the third week, and sometimes at the beginning of convalescence; it causes no recrudescense nor return of fever.
5. Puerperal accidents are the exception.
6. The immediate causes of abortion are unknown; elevated temperature, active or passive uterine congestion, and changes in the blood, although seemingly the most probable, cannot be regarded as the causes in all cases.
7. The treatment for the fever and the miscarriage is the same as for each condition alone.—*Med. World.*

**Dr. J. M. DaCosta** has been testing the therapeutic value of the salts of nickel. The sulphate proved of some value in obstinate diarrhœa. The bromide, however, is the most valuable of all, and will probably take a permanent place in the materia medica. Its action is similar to the other bromides, but a much smaller dose suffices. Five to seven and a half grains is an average dose, and ten grains is a decided one. It relieves congestive forms of headache and quiets the system generally. In epilepsy it does quite as well as other bromides, but, as above mentioned, a much smaller dose suffices.—*Med. World.*

**Antagonism between Syphilis and Vaccine.**—The Drug. Cir. and Chem. Gaz. for May, 1883, says that Dr. Polin inclines to the belief that there is an antagonism between the vaccine virus and that of syphilis. He was led to this view by the results of some vaccinations performed by him in Algeria. Of 471 children, the vaccination was successful in 410, all of whom were free from any syphilitic taint. Of the 61 children in whom the inoculation did not succeed, 48 presented indubitable evidences of syphilis. (We have made the same observation.—EDS.)—*N. Y. Med. Times.*

**Chloral in Albuminuria.**—In a bad case of albuminuria, in which the urine had been largely impregnated with albumen, the specimens passed after taking the chloral were of average specific gravity 1016, acid with no albumen; specimens passed at other periods contained albumen and granular and hyaline tube-casts.

A continuation of the chloral treatment resulted in complete disappearance of albumen from the urine, and with it disappearance of the other symptoms I have mentioned. In the middle of the month of July she had so far recovered that she was able to be removed to the sea-side. At the present time she is better than she has been for many months past, and, with the exception of amenorhœa, she is quite well. No explanation is offered as to how the chloral was followed by such beneficial results. Suffice it to say that under its use, a lady so prostrate that she could not stand, with a dilated heart, albuminuria, and marked œdema of feet and legs—indications of a grave constitutional state—has simply been rescued from death. The chloral did not produce any apparent diuresis or diaphoresis.

**Amenorrhœa.**—Dr. Murrell, in Lancet, says: A married woman came to me complaining that she was never regular. She had had nine children in as many years, and rarely saw anything more than once between her pregnancies. She had been suckling for eight months, and had not been poorly for seventeen months—the nine months she had carried and the eight months she had suckled. She was not in a family:way, but said she expected she would be soon if she weaned the baby. She did not know when she ought to be poorly, and had given up all expectation of seeing anything. She was ordered two one-grain permanganate of potash pills four times a day, and came on poorly a fortnight after, the first time for seventeen months.

**An Adjuvant to Chloroform.**—M. Daster experimented to increase the effect of chloroform, while at the same time he diminishes the danger by preceding its administration by a hypodermic injection of the following mixture:

Aq-destil, 10 grams.

Muriate of morphine, 10 centegrams.

Sulph-atropine, 5 milligrams.

One gramme of this injected prior to chloroformization, a very calm anæsthesia is brought about.—*Gaz. Med. de Nanets.*

**Acute Goitre.**—Surgeon-Major Gore, Edinburgh Med. Journal, records 30 cases of cure of this disease among the soldiers of a native Indian regiment, by biniodide of mercury, rubbed in for ten minutes or more, as the patient sat with the enlarged gland exposed to the sun or a strong fire. In some of the cases the swelling had been observed for about ten days before treatment. Only one case was any length of time in the hospital, viz: 79 days; an anæmic man, aged 22 years. The average duration of the treatment was 22-6 days.—*Jour. Ame. Med. Ass'n.*

**Organic Matter in Drinking-Water.**—A trustworthy writer aptly remarks, those who are familiar with the most recent sanitary experience realize that it is the *quality* rather than the *absolute quantity* of organic matter that is the most important factor in the sanitary judgment of a drinking-water. A water which contains a large amount of one kind of organic substance may be much more wholesome, or far less unwholesome, than that which contains only a small amount of another kind. It is a matter of actual experience that a water, notwithstanding it contains a large amount of nitrogenous organic matter capable of yielding albuminoid ammonia, may be found to be practically wholesome, or at least may be drunk for a long period without apparently producing any injurious effects; while, on the other hand, a water which contains *even a minimum* of organic substances capable of yielding albuminoid ammonia may nevertheless contain or develop the *materies morbi*, or unknown causal "something" of a specific disease.

**The Ownership of Prescriptions in France.**—A rather curious lawsuit lately took place before the justice of the peace at St. Germain, in which a pharmacist was prosecuted for having refused to give up a prescription that was taken to him by a patient. The patient claimed the prescription as being his property, which the druggist contested; but the tribunal decided otherwise, and the prescription was restored to the patient. The Societe de Medecine Legale, commenting upon the case, admitted that the decision of the judge was unassailable from a legal point of view, but took the same ground as most medical societies in this country, viz., that the repetition of prescriptions is sometimes injurious to patients, and always detrimental to physicians, in a pecuniary sense.—*Druggists' Circular and Chemical Gazette.*

**Paraplegia in a Child caused by Intestinal Worms.**—A child of ten years was brought to the London Hospital and placed under the care of Dr. Thorowgood as a case of infantile paralysis. She had lost strength in her lower limbs gradually, for three months had been unable to walk, and was then quite unable to stand. The legs were not much wasted. Upon inquiry, it was ascertained that the patient also had been troubled with ascarides. An aloetic purgative mixture was given for several days, which acted well and brought away many worms. In two days she was able to run and walk about the ward as well as could be desired; and a few days later, as she seemed perfectly well, she was allowed to go home.—*Lancet, July 28.*

**In** the Journal de Medicine de Paris are collected the results obtained by several observers in the prevention of abortion and premature labor by assafetida. In ninety per cent. of the cases so treated the patients (who had aborted from two to five times in former pregnancies) went on to full term.—*Louisville Med. News.*

**Prevention of Mammary Abscess.**—A mixture of chloroform and glycerine, well shaken and quickly applied, and covered with oiled silk, is highly recommended.—*Drug. Circular.*

**Croton-Chloral in the Treatment of Whooping-Cough.**—W. C. Webb, M.D., of Bryantsville, Ky., ("American Practitioner," August, 1883), has come to the conclusion, from the treatment of nearly two hundred cases of whooping-cough, that croton-chloral is by far the most valuable single remedy for its relief. He has found that it is well borne by children. To affect the disease it must be given in decided doses. A child 12 months old will bear one grain of the medicine every four hours throughout the twenty-four. During the first week not less than this should be given. Thereafter the cough is usually so much relieved that few if any doses are required at night If the drug be thus pushed to its full effect, there are few cases that may not be entirely controlled in a fortnight. The dose for children ten years old should be two grains every four hours; adults will bear only about four-grain doses. The drug thus used does not derange digestion or affect the vital nervous centers. The first few doses may cause some irritation about the throat and fauces, but this soon ceases. The relief is so marked in some cases that patients fall asleep in their chairs.

Croton-chloral, if pulverized, will dissolve readily in compound tincture of cardamom. The following is a good prescription—

℞. Croton-chloral .................................. ʒ i,

Tinct. cardamo ................................ } aa. ℥ ij.

Glycerin ........................................ }

M. Sig. One-half teaspoonful every four hours, for a child two years old and under.

A less expensive and very useful mixture is as follows:

℞. Croton-chloral .................................. ʒ i,

Tinct. belladonna ................................ ʒ ij,

Tinct. cardam. co ................................ ℥ ij,

Glycerin ........................................ ℥ iij.

M. Sig. Dose, one-half teaspoonful.

If the paroxysms of cough are exceedingly severe, and if there is extreme gastric irritability, the croton-chloral should be preceded by a few whiffs of chloroform. The anæsthetic thus used produces the happiest effects, and it need not be repeated more than two or three times. The combination of bromides with the croton-chloral is of doubtful utility. If any of them are to be used, the bromide of quinine should be preferred. Watchfnlness should, of course, be exercised during the use of croton-chloral, lest toxic symptoms should appear.—*N. Y. Med. Record.*

**Iodo-Ferrated Syrup of Coffee.**—Povesi gives the following formula for an iodo-ferrated syrup of coffee, which is especially adapted, from its agreeable flavor, for use in cases of scrofula occurring in children: ℞ Syr. ferri. iod., syr. coffee, aa partes æquales. The dose is one-third that of syr. ferri. iod.—*Gaellara's Med. Journal.*

